# Nucleosome rotational setting is associated with transcriptional regulation in promoters of tissue-specific human genes

**DOI:** 10.1186/gb-2010-11-5-r51

**Published:** 2010-05-12

**Authors:** Charles Hebert, Hugues Roest Crollius

**Affiliations:** 1 Dyogen Group, Institut de Biologie de l'Ecole Normale Supérieure (IBENS), 46 rue d'Ulm, CNRS UMR8197, INSERM U1024, 75005 Paris Cedex 05, France

## Abstract

**Background:**

The position of a nucleosome, both translational along the DNA molecule and rotational between the histone core and the DNA, is controlled by many factors, including the regular occurrence of specific dinucleotides with a period of approximately 10 bp, important for the rotational setting of the DNA around the histone octamer.

**Results:**

We show that such a 10 bp periodic signal of purine-purine dinucleotides occurs in phase with the transcription start site (TSS) of human genes and is centered on the position of the first (+1) nucleosome downstream of the TSS. These data support a direct link between transcription and the rotational setting of the nucleosome. The periodic signal is most prevalent in genes that contain CpG islands that are expressed at low levels in a tissue-specific manner and are involved in the control of transcription.

**Conclusions:**

These results, together with several lines of evidence from the recent literature, support a new model whereby the +1 nucleosome could be more efficiently disassembled from gene promoters by H3K56 acetylation marks if the periodic signal specifies an optimal rotational setting.

## Abstract 2

Human genes contain a 10 bp repeat of RR dinucleotides focused around the first nucleosome position suggesting a role in transcriptional control.

## Background

Nucleosomes, composed of 147 bp of DNA wrapped around a histone octamer, play a fundamental role of compacting DNA molecules inside the nucleus of eukaryotic cells [1], but also in the regulation of gene expression [2,3]. Elucidating the molecular mechanisms that specify the position of nucleosomes in a genome is important to understand their role at the crossroads of essential cellular functions.

Factors influencing nucleosome positioning likely include DNA sequence-based information (either to specify a favorable or unfavorable DNA structure or to allow for DNA-histone interactions), contacts between neighboring nucleosomes, and chromatin remodeling proteins. The extent and the modalities of these contributions are still being investigated, and different models have been proposed to explain whole genome nucleosome mapping data in different organisms [4-7]. These results, while primarily focusing on the translational positions of nucleosomes along the DNA molecule, also show that the rotational position of the histone octamer with respect to the DNA molecule is important. High-resolution maps indicate that individual nucleosomes tend to settle at approximately 10-bp intervals around an average position in the genome [4,6,8]. Histone cores, when forming a nucleosome with the DNA, thus appear to locally select one of several alternative positions on the DNA, as long as they are separated by distances multiple of a helical turn. Importantly, selecting one position rather than the next will translate the nucleosome by 10 bp, but will not change the rotational angle of the histone core with respect to the DNA molecule and its molecular environment. To wedge histones in their preferred rotational setting, the main theoretic constraint is a periodic occurrence of specific dinucleotides at approximately 10-bp intervals in phase with nucleosome positions [9-11]. This signal is significantly different between species. In yeast, it has been characterized as periodic frequencies of dinucleotides containing only adenine and/or thymidine (WW dinucleotides), with antiphased periodic frequencies of dinucleotides containing cytidines and/or guanines (SS dinucleotides) [12]. In mammalian genomes, the most consistent 10-bp periodic signal is composed of periodic purine dinucleotides (A or G, abbreviated RR), with antiphased pyrimidine dinucleotide frequencies (C or T, abbreviated YY) [13-16], although other combinations of di- and trinucleotides have also been observed [17,18].

In yeast, high resolution mapping of nucleosomes containing the H2A.Z histone variant, which is typically found in nucleosomes flanking the transcription start site (TSS) of genes [19,20], led to a model where this rotational setting could be important to present the histone H3 tail in a favorable position at the promoter, or to expose transcription factor binding sites at the nucleosome surface [4]. In the human genome, a high-resolution map of H2A.Z nucleosomes recently led to the conclusion that, in contrast to the yeast genome, a pronounced 10-bp periodicity of specific dinucleotides is absent [8] near the TSS. Here we examine sequences flanking human TSSs, and we find that a 10-bp periodicity of the same magnitude as that seen in yeast, but of RR rather than WW dinucleotides, does coincide with the first nucleosome after the TSS (+1 nucleosome). Importantly, the signal is specifically in phase with the TSS, suggesting a direct link between transcription and the +1 nucleosome. We analyze the periodic signal with respect to CpG island density, gene expression level and breadth, gene functional annotations, and histone modification marks. We conclude that the periodic signal is likely to play a role in setting the rotational angle of the histone core in the +1 nucleosome, and we propose a model where nucleosome interacting proteins, such as the EP300 histone acetylase, may efficiently trigger histone disassembly prior to RNA polymerase II (RNA pol II) elongation if the rotational setting of the nucleosome is optimal.

## Results

### A periodic dinucleotide frequency in phase with the TSS coincides with +1 nucleosomes

**Figure 1:**
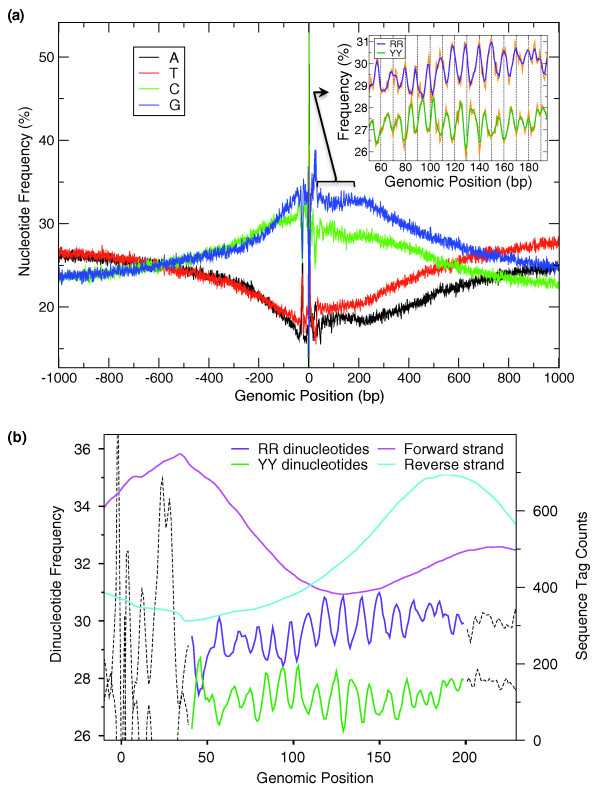
**A 10-bp periodic signal is present downstream of human transcription start sites**. **(a) **Average compositional profiles around 13,622 human promoters. A 1,000-bp region on either side of each TSS was extracted from the genome and the 13,622 sequences were aligned at the TSS (base +1 is the first transcribed base). The average composition at each base-pair position is shown on the y-axis. Inset: average compositional profile of purine-purine and pyrimidine-pyrimidine dinucleotides between positions +40 and +200. The raw signal is shown in orange and a 3-bp smoothed distribution is shown in purple (RR) and dark green (YY). **(b) **DNA sequences of the +1 nucleosome contain the periodic signal. Sequence tags from nucleosome-bound DNA obtained by a ChIP-seq experiment [23] were remapped to the human genome and their density was smoothed with a sliding 70-bp window (see Materials and methods). Tags mapped to the forward (magenta) and the reverse (cyan) strand mark the 5' and 3' ends of nucleosome bound DNA fragments, respectively. Counter-phased RR (purple) and YY (green) dinucleotide frequencies, and base pair coordinates are as in (a).

Approximately 30,000 human TSSs have previously been identified experimentally by oligo-capped cDNA sequencing [21]. From these, we selected a subset of 13,622 well-supported and non-overlapping TSSs (see Materials and methods) and aligned them at the position of the first transcribed base. The average nucleotide composition profile displays the characteristic pattern of human promoters, with a progressive increase in GC content around the TSS due to the concentration of CpG islands, and two sharp peaks of TA and YR nucleotide bases at positions [-32:-27] and [-1:+1] due, respectively, to the TATA box and the initiator sequence (Figure 1a). Notably, the frequency of C versus G decreases after the TSS, while the frequency of T versus A increases, as previously described in the context of transcriptionally induced mutational biases [22].

After the TSS, the frequencies of both G and C remain elevated for approximately 200 bases, thus forming a plateau, before slowly decreasing. Closer examination of the nucleotide composition across the plateau reveals a striking pattern of oscillating frequencies of all four nucleotides, with A and G in phase, and C and T shifted by 5 bp in counter phase (Figure S1A in Additional file 1). The period of the regular pattern is approximately 10 bases and the purine nucleotide peaks are separated from the TSS by a distance multiple of 10 bases, thus residing on the same side of the DNA double helix as the TSS. To better characterize the signal, we analyzed the period of the 16 possible dinucleotide frequencies using discrete Fourier transform (DFT; Figure S1B in Additional file 1; see Materials and methods) and found that mainly purine-purine (RR) and pyrimidine-pyrimidine (YY) dinucleotides contribute to the periodic signal (Figure 1a, inset) in phase and counter-phase, respectively, with the TSS. Randomly shifting the sequences by 1 to 9 bases relative to the TSS completely abolishes the signal (average power spectral density (PSD) magnitude at 10 bp = 0.015; *P*-value = 2.2 × 10^-16^, Wilcoxon rank sum test).

If this signal is linked to nucleosome positioning, it should coincide with experimentally defined nucleosome positions from genome-wide mapping efforts. To verify this, we realigned the sequence tags from a recent ChIP-seq experiment aiming at defining the positions of all nucleosomes in human CD4+ cell lines [23], and we focused on the region immediately downstream of the TSS positions used in our study. Remarkably, the 5' ends of the sequence tags of the forward and reverse strands from the ChIP-seq experiment, which define the boundaries of the nucleosome-bound DNA, show maximal densities that precisely flank the periodic signal (Figure 1b). Thus, DNA sequences of +1 nucleosomes immediately downstream of human TSSs display periodic purine-purine (RR) and pyrimidine-pyrimidine (YY) frequencies.

### The periodic signal is correlated with CpG islands

Despite our attempts, the periodic RR and YY signal cannot be detected in individual sequences beyond those periodic dinucleotides one would expect by chance alone, even using standard autocorrelation analysis (data not shown). This lack of significant periodic dinucleotide patterns in individual human H2A.Z sequences has been noted previously using autocorrelation analysis, in contrast to yeast nucleosomal sequences, where periodic patterns appear readily [8] using these approaches. However, a more sensitive autocorrelation analysis, called autocorrelation spectral estimation, recently showed that 10- and 11-bp periodic AA/TT dinucleotide signals exist in human nucleosomal sequences, while the 11-bp signal is specific to the regions flanking the TSS [24].

**Figure 2:**
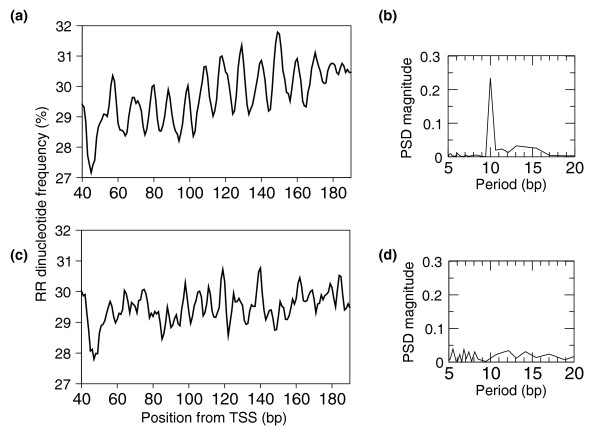
**CpG islands separate transcription start sites with and without the 10-bp RR periodic signal**. **(a, b) **The 9,622 TSSs associated with a CpG island show a clear periodic signal (a) that translates into a strong and specific 10-bp periodic signal after DFT analysis (b). **(c, d) **In contrast, the 3,978 TSSs without CpG islands do not display an obvious periodic pattern (c), with no associated distinctive signal after DFT analysis (d).

Together, the fact that a periodic signal in the region following the TSS can only be measured using either sensitive autocorrelation measures on individual sequences [24] or the average dinucleotide frequencies of a large set of sequences (this study) suggests that, in contrast to yeast, the RR/YY dinucleotides in human show only a weak periodicity at the level of individual sequences. We thus resolved to use large sets of sequences by partitioning the TSSs into classes according to properties conventionally used to describe genes and to examine if the signal concentrates in a subset of promoters. CpG islands [25] are featured in a majority of mammalian genes as a consequence of the hypomethylation of cytosine in CpG dinucleotides in the germ line. To identify CpG islands in the 13,622 promoter sequences, we applied a parameterized Gaussian mixture model (see Supporting information and Figure S2 in Additional file 1) that has been shown to be more reliable than using *ad hoc *length and frequency thresholds [26]. We found that 9,644 promoters are associated with a CpG island (70.8%) while the remaining 3,978 promoters (29.2%) show similar levels of CpG dinucleotides as the rest of the genome. Strikingly, promoters with CpG islands show a stronger periodic signal than the complete population of 13,622 promoters, while those without CpG islands do not show any periodicity of RR/YY dinucleotides (Figure 2).

In each group of promoters, we performed a DFT analysis on each of the 16 dinucleotide average frequency profiles between positions +40 and +190 after the TSS (Figure S3 in Additional file 1). A differential comparison between the sets of promoters with and without CpG islands (Supporting information in Additional file 1) should identify those dinucleotides that contribute most to the periodic pattern. Interestingly, in CpG island-containing promoters, GA and AG rank highest among RR dinucleotides, and their complementary CT and TC rank highest among YY dinucleotides (Table S1 in Additional file 1). Notably, dinucleotides AA, TT and TA, which show strong periodic patterns in yeast nucleosome-bound DNA [4,12], do not contribute to the periodic pattern seen here in human CpG-containing promoters. Within promoters with CpG islands, the strength of the periodic signal is not, however, correlated with the overrepresentation of CpG dinucleotides (Supporting information in Additional file 1).

### The periodic signal is most prevalent in tissue-specific genes involved in transcription control

**Figure 3:**
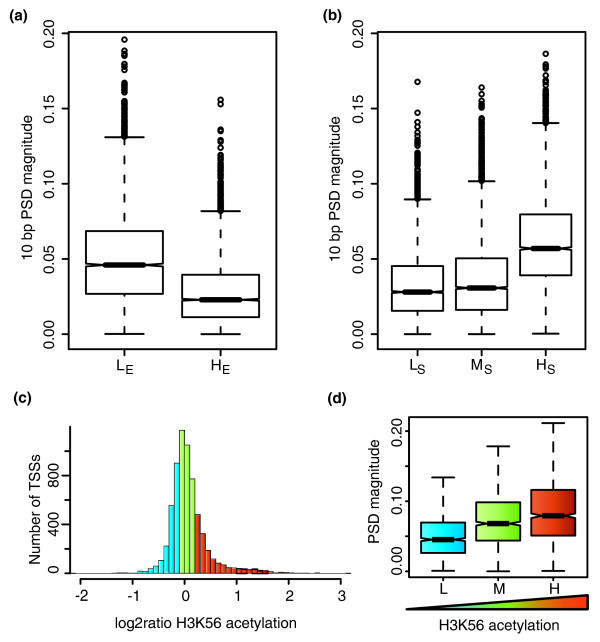
**The periodic signal varies with expression level and specificity, and H3K56 acetylation**. **(a) **We divided 4,372 genes into two groups (low expression (L_E_) and high expression (H_E_)) according to their median expression level across 72 tissues. The boxplots show the distribution of the magnitude of the 10-bp periodic signal for 5,000 bootstrap iterations on 1,000 randomly selected TSSs in each group (see Materials and methods). The 10-bp periodic signal is stronger in the low expression group than in the high expression group. **(b) **The same set of genes were divided into three groups according to their tissue specificity (low, medium and high tissue specificity) and the same bootstrap analysis was performed. **(c) **The distribution of the normalized H3K56ac enrichment (log2 ratio) for the 6,518 TSSs that possess an H3K56ac sequence tag (see Materials and methods) is shown. The TSSs were divided into three groups of equal size with, respectively, low (L, blue) medium (M, green) and high (H, orange) H3K56ac enrichment ratios. **(d) **The three groups of H3K56ac enrichment are associated with different strengths of the periodic RR/YY signal. A randomization test shows that increased H3K56 acetylation levels is significantly correlated with increased 10-bp periodic signal (Wilcoxon rank sum test, one sided: L versus M *P*-value = 2.2 × 10^-16^; M versus H *P*-value = 3.8 × 10^-07^; L versus H *P*-value = 2.2 × 10^-16^).

Because the periodic pattern is evident only when promoters are aligned to their TSS, properties related to gene transcription may be correlated with the strength of the signal. We partitioned the 9,644 TSSs with CpG islands into two groups with, respectively, low (L_E_) and high (H_E_) median expression levels in 72 non-cancerous tissues (see Materials and methods) and measured the distribution of the magnitude of the 10-bp RR periodicity for each group (Figure 3a). TSSs associated with lower expression levels (L_E _group) show significantly stronger periodic signals than TSSs with high expression values (*P*-value = 2.2 × 10^-16^, Wilcoxon rank sum test). When genes are partitioned according to their tissue specificity (see Materials and methods), genes with high tissue specificity (H_S_) show a significantly stronger periodic signal than genes that are more broadly expressed (medium (M_S_) or low (L_S_) tissue specificity; L_S _or M_S _group versus H_S _group *P*-value = 2.2 × 10^-16^, Wilcoxon rank sum test; Figure 3b). In line with this, genes from the H_S _group also show a reduced expression level compared to genes of the L_S _or M_S _group (*P*-value = 2.0 × 10^-16^, Wilcoxon rank sum test). Compared with the L_S _group, the H_S _group is also enriched in Gene Ontology terms associated with DNA-dependent transcription, and the regulation of transcription (Methods and Table S2 in Additional file 1). The enrichment for DNA-dependent transcription is mainly due to an excess of genes coding for transcription factors. Thus, genes with lower expression levels and high tissue specificity coding for proteins involved in transcription regulation show a stronger periodic RR and YY dinucleotide frequency in phase with their TSS and overlapping the first nucleosome in the transcribed sequence.

### EP300 activity is correlated with increased periodic RR/YY dinucleotides

Genes coding for tissue-specific transcription factors are themselves highly regulated, and given their significant association with a nucleosome rotational positioning signal, we hypothesized that the control of their transcription and information carried by the first nucleosome are somehow connected. Histone modifications are obvious candidates for this potential connection. Histones transiently harbor acetylation and methylation marks deposited by chromatin-modifying enzymes recruited by a diverse array of proteins. One such modifying enzyme is EP300, which directly associates with the pre-initiation complex that includes RNA Pol II [27], and also binds DNA at a known consensus sequence [28]. EP300 is known to acetylate histones at the following sites: H3K14, H3K18, H4K5, H4K8, H2AK5, H2BK12, H2BK15 [29]. Of these seven marks, six were recently part of a genome-wide mapping of histone modifications in human CD4+ cells [30]. We first tested for the presence of EP300 DNA binding sites in the 13,622 TSSs studied here, and found that they are significantly associated with genes where the first nucleosome carries at least one of the six acetylation marks (*P*-value = 3 × 10^-5^, randomization test), in line with expectations. Second, we also searched for the EP300 DNA binding site in all 13,622 TSSs independently of their histone modification status and found that it is significantly associated with the periodic 10-bp RR frequency signal (*P*-value = 1 × 10^-3^, randomization test). Third, the intensity of histone acetylations by EP300 on the first nucleosome, as measured by the ChIP-seq sequence tag counts, is also correlated with an increasing magnitude of the periodic signal (*P*-value = 2 × 10^-15^, Pearson correlation test; Figure S4 in Additional file 1). Most strikingly, this is also verified for an acetylation mark recently attributed to EP300 on H3K56 [31], in the globular domain of histone H3. Using recent ChIP-chip results obtained using H3K56ac in the human genome [32], we show here that the level of H3K56 acetylation is correlated with an increased 10-bp periodicity (Figure 3c, d; low H3K56ac enrichment ratio group versus high H3K56ac enrichment ratio group *P*-value = 2.2 × 10^-16^, one-sided Wilcoxon rank sum test). This evidence strongly supports the above hypothesis that a histone-modifying enzyme such as EP300 involved in the first steps of transcription elongation may require a specific rotational setting of the first nucleosome to efficiently carry out its functions (see Discussion).

### Conservation of the periodic signal in eukaryotic genomes

The periodic signal observed here appears to be universally present in eukaryotes, albeit involving different dinucleotides. The same periodic RR/YY dinucleotide frequency is seen in human and mouse promoters, but interestingly the medaka fish *Oryzias latipes *displays a strong periodic signal contributed by AA and TT dinucleotides downstream of the TSS, similar to yeast (Figure S5 in Additional file 1). In yeast, however, the periodic signal appears shorter and is immediately downstream of the TSS [33], instead of being shifted to the +40 position as in vertebrates.

## Discussion

We describe here a new 10-bp periodic signal present downstream of human TSSs that is concentrated in genes that possess CpG islands, that are expressed at low level in a tissue specific pattern, and that are enriched in functions related to transcription control. Importantly, the signal is centered over the position of experimentally mapped nucleosomes. This result contrasts with a recent study describing the mapping of H2A.Z-containing nucleosomes in the human genome, which concluded that such a periodic signal is essentially absent in human promoters, whereas it had been previously observed in yeast [8]. However, this former study aligned promoters on the predicted +1 nucleosome dyad position, not on experimentally annotated TSSs as here. Tolstorukov *et al*. [8] discuss the possibility that a periodic dinucleotide profile may arise in the average frequencies of a set of sequences, even if the periodic signal is not directly related to nucleosome positioning. Such a signal may occur if, for example, a short motif has strong nucleosome positioning properties, but would still allow the histone core to shift by a few base pairs along the sequence to settle in the most favorable configuration in terms of deformation energy cost. Once sequences are obtained by the ChIP-seq technology and aligned at the dyad, their average nucleotide profile may theoretically show such a periodic pattern as a consequence of nucleosome rotational positioning rather than as a cause. Here, however, we align nucleosome sequences independently of the ChIP-seq technology, using the TSS as sole reference. The above scenario may only be applicable to our data if a strong nucleosome positioning motif is itself aligned to the TSS, unrelated to the periodic pattern which, in this case, would be secondary to the motif. Even under this non-parsimonious scenario, however, the conclusion that the rotational setting of the nucleosome is linked to the TSS remains unchanged.

Our work thus underlines a tight coupling between the periodic signal and transcription. We show that the strength of the periodic signal can be correlated with promoters that contain EP300 binding sites, and histones of the +1 nucleosome that are acetylated at residues known to be targets of EP300. Based on these results, we propose a theoretical model that explains how EP300 may efficiently trigger transcription elongation in genes that require rapid and coordinated expression.

**Figure 4:**
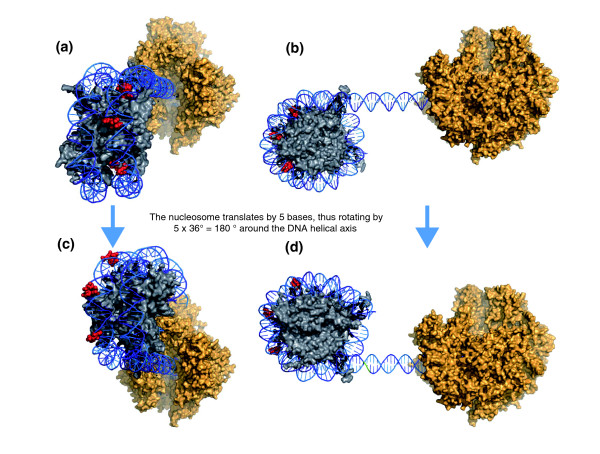
**Schematic representation of the spatial relationships between the nucleosome, the DNA molecule and RNA Pol II**. **(a) **The nucleosome histone core (grey) is positioned on the DNA molecule (blue) with the first three minor groove-histone contact points containing RR dinucleotides (red). The RNA Pol II complex (gold) is shown here without its associated co-factors for clarity. **(b) **The same as in (a) but a side view, showing the RR dinucleotide in intimate contact with the histones. **(c) **If the nucleosome is shifted 5 bases closer to the RNA Pol II, it must rotate in space by 5 × 36° = 180° around the helical axis with respect to RNA pol II in order to preserve the contacts between the histones and the minor groove. **(d) **The same as in (c) but a side view, showing how the RR dinucleotides are now facing outwards and how the RNA Pol II 'sees' the first nucleosome from an entirely different angle.

EP300 was recently found to acetylate lysine 56 of histone H3 (H3K56) in human and *Drosophila *[31], a modification that promotes nucleosome disassembly during transcription [34] in yeast. Instead of residing on histone tails, as for many acetylation and methylation targets, H3K56 is located on the globular histone core [35,36], a location that restricts its accessibility to EP300. As an additional source of spatial constraint, EP300 interacts with unphosphorylated RNA pol II [37] and binds DNA, and is likely to be subject to one or both of these interactions while depositing an acetylation mark on H3K56. EP300 is therefore unable to freely move on its histone target. To remain efficient, it is reasonable that this important step in the elongation phase of RNA pol II transcription must be spatially optimized. We propose that RR and YY dinucleotides located at key positions in the DNA sequence wrapped around the histone core may be the information required to position the nucleosome at the optimal spatial coordinates for EP300 interaction. Indeed, histones interact with DNA in regions where the minor groove of the double helix faces inwards. If the nucleosome shifts its position by 1 bp, it must rotate by approximately 36° around the DNA helical axis in order for histones to remain in contact with the minor groove. If the nucleosome shifts by a full 10.2- to 10.5-bp helical turn, it completes a 360° circular motion around the helical axis. The spatial positioning of the nucleosome with respect to the DNA molecule and its associated protein complexes is thus precisely dependent on its local position, at single base pair resolution (Figure 4).

**Figure 5:**
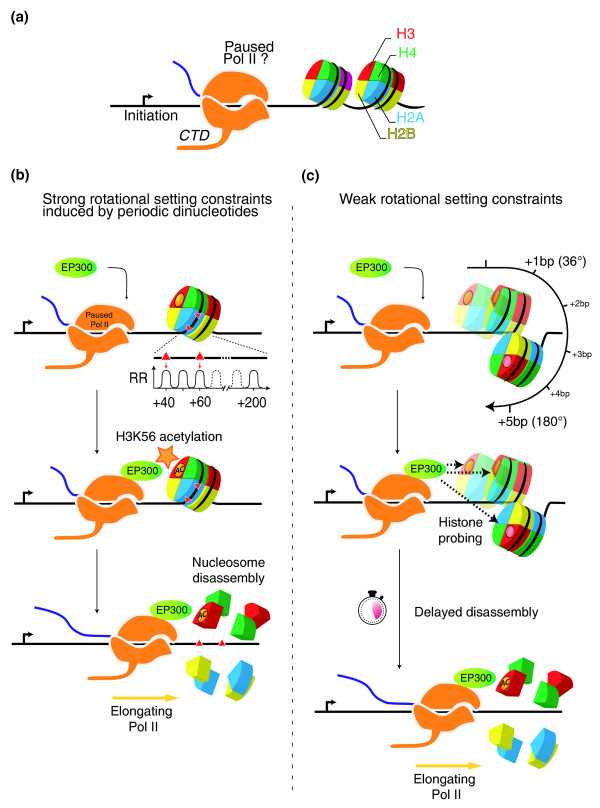
**A theoretical model of how the rotational setting of a nucleosome may facilitate its own disassembly by EP300 acetylation**. **(a) **RNA pol II (Pol II) after transcription initiation at the TSS (black arrow). Our model is consistent with Pol II that is paused at this stage, although this is not a requirement. **(b, c) **Subsequent steps leading to elongation if the nucleosome is rotationally constrained (b), and the process for fuzzier nucleosome positioning (c). In (b), red triangles indicate the positions of two RR dinucleotides at a distance multiple of 10 bp from the TSS. Several hundred promoters carrying such a signal in the human genome would generate the pattern shown in Figure 2a. On a given sequence, this may be sufficient to constrain the +1 nucleosome to remain set at a specific position and thus at a specific rotational angle with respect to the advancing Pol II. After binding to its DNA recognition site and/or being recruited by other proteins, EP300 binds to Pol II and is now optimally located in space to deposit acetylation marks on the +1 nucleosome. These may include several targets on histone tails but critically includes H3K56 located on the globular part of H3 (orange circle), required for tipping the nucleosome assembly/disassembly equilibrium towards disassembly. Next, Pol II is free to engage in the elongation phase. In (c), RR dinucleotides occur randomly in the sequence and the +1 nucleosome may therefore adopt any rotational angle. Shown here are three possible nucleosome locations (+0, +1 and +5 bp from the position shown in (b)), each with a different angle. For instance, a 5-bp shift equivalent to half the helical pitch would rotate the nucleosome by approximately 180° with reference to the position at +0 bp, as shown in Figure 4. Depending on the nucleosome angle, EP300 is not optimally located with respect to its target and needs to search or probe for its histone target, thus delaying H3K56 acetylation and subsequent nucleosome disassembly.

It is tempting to link our model to the phenomenon of RNA Pol II 'pausing' after transcription initiation [38,39]. RNA pol II pausing is thought to poise the polymerase for transcription, enabling rapid induction of the elongation phase, upon receiving the appropriate signal. This requires, amongst other processes, that the histone core be removed from the DNA molecule, and strikingly, H3K56 acetylation is thought to be a determining factor in tipping the nucleosome assembly/disassembly equilibrium towards disassembly [34]. Our model therefore predicts that the periodic signal may be a mechanism by which genes that need rapid activation of the elongation phase after RNA Pol II pausing may expedite nucleosome disassembly by efficiently acetylating H3K56. Indeed, it may be expected that genes poised for rapid expression through RNA Pol II stalling would be subjected to a following step that is also optimized for its efficiency (Figure 5). This model offers a possible mechanism for the release of the paused Pol II, after its conversion to an elongation-compatible form by P-TEFb [40,41]. Remarkably, our model also provides a possible explanation for the somewhat counterintuitive observation that genes harboring elongating Pol II show well-positioned +1 nucleosomes [23]. Indeed, a +1 nucleosome that is in phase with a rotational positioning signal will show little translational variability in mapping experiments yet will be efficiently disassembled to make way for Pol II elongation. Our model also explains the observation that Pol II appears to pause primarily at 20, 30 or 40 bp from the TSS, that is, at positions that are multiples of 10 bp [23,42,43]. Indeed, if the nucleosome itself is resting at positions that are distant from the TSS by such a unit length, then the abutting RNA Pol II would be tied to the same positional constraints. Finally, our model predicts that modulating the rotational orientation of a nucleosome may be an efficient mechanism to regulate gene activation, in a way that is epigenetically heritable. In such circumstances, chromatin remodeling factors would promote the shifting of the histone core by a few base pairs from an unfavorable to a favorable orientation and back, thus controlling the potential for H3K56 acetylation and nucleosome disassembly. The fact that the SWI/SNF complex is required to stimulate transcription elongation in mammalian cells [44] by remodeling the +1 nucleosome is consistent with this prediction. It would be interesting to compare our model based on human TSS sequence analysis to the situation in *Drosophila*, where more experimental data are available. Currently, the precision of annotated TSSs in the *Drosophila *genome is not sufficient to allow the identification of a periodic signal as described in yeast or human, although this is likely to change in the near future.

A different model was recently proposed to account for H2A.Z-related dinucleotide periodicities near the yeast TSS [3,4]. In this model, the preferred rotational setting exposes transcription factor binding sequences on the surface of the nucleosome that would otherwise be facing the histone core. Binding of transcription factors would play a role in regulating the translational displacement of the nucleosome, which may be important for gene activation. While our findings are not incompatible with this model developed in yeast, we did not find evidence for specific periodic transcription factor binding site occurrences downstream of human promoters (Supporting information and Figures S9 and S10 in Additional file 1).

Several observations may explain why one or several RR/YY dinucleotides placed at positions separated by multiples of 10 bp along the wrapped DNA can direct the histone core to settle in a specific position and thus specify the rotational setting of the nucleosome. These include: strong stacking interactions between purines facilitating the collapse of the minor groove, and weaker interactions between the complementary pyrimidines facilitating their deformation in the major groove [15]; the GG = CC and AG = CT steps are, of all steps, the only two that form cross-chain hydrogen bonds in the minor groove, which is probably a determinant of the energetically more favorable smooth versus kinked bending of the DNA [10]; and an arginine side-chain is located in the minor groove of all histone-DNA binding sites except for one, where the potential discriminator for direct read out is the adenine C2 group versus the guanine N2 group [45] (Figure S8 in Additional file 1). However, any structural explanation for the RR/YY periodicity in human and mouse should account for the fact that different eukaryotic species appear to rely on different combinations of dinucleotides in the periodic signal.

## Conclusions

The RR and YY periodic signals described here suggest a new model where sequence information is directly exploited to create an optimal spatial topology between at least three entities: the RNA Pol II associated with cofactors and EP300, the DNA molecule and the +1 nucleosome (Figure 5). The convergence of many observations leading to this model is striking, yet it is possible that EP300 and nucleosome rotational orientations are not mechanistically linked as suggested, because EP300 activity may be linked to CpG island-containing TSSs due to their role as transcriptional co-activators. Our ability to design experiments that would directly test the model is limited because we currently lack a good understanding of the structural basis for the rotational preference for specific dinucleotides. In particular, we do not know the minimal number of RR (or YY) dinucleotides in phase with the TSS that would be required to specify this spatial topology, but the model nevertheless suggests that if mutations eliminate the crucial RR (or YY) dinucleotides, elongation may not proceed with the required efficiency and may decrease the expression of the gene, thus potentially causing abnormal phenotypes.

## Materials and methods

### Transcription start site database

All TSSs were extracted from the DBTSS database version 6, 15 September 2007 [21]. In case TSSs were within 200 bp of each other, we considered the most frequent only. TSSs supported by less than two cDNAs mapping to the exact same position were not considered. Each TSS was mapped to the NCBI36 human genome assembly and assigned to the nearest Ensembl gene (version 49). The final dataset contains 13,622 TSSs associated with 12,028 Ensembl genes.

### Power spectral analysis

We applied DFT to compute the PSDs or 'periodograms' of the periodic signals using R and Python/Numpy functions. The periodogram magnitude is the squared modulus of the Fourier coefficient divided by the length of the series. Each PSD area is normalized to 1 before extracting the magnitude of the periodicity at 10 bp. To reduce the noise caused by the small size of the genomic region over which the measures are performed (+40 to +190 after the TSS), we applied a 3-bp smoothing window and multiplied the signal with a Hamming window prior to the DFT analysis.

### Alignment to the transcription start site

To test the specificity of the phasing of the signal to the TSS, regions from position +40 to +190 where extracted from all 13,622 sequences and a random number (between 1 and 9) of bases was added at their 5' end to introduce a random shift. The average RR frequency was then measured at each position and used to compute the PSD magnitude at 10 bp. The process was repeated 500 times to obtain a distribution, which was compared to the PSD magnitude at 10 bp of the compositional profile of the real sequences (without shift).

### ChIP-seq and ChIP-chip data

Nucleosome tags [23] were downloaded from the NCBI Short Read Archive (SRA) repository under accession number [SRA:SRA000234]. We considered only the human activated CD4+ T cell experiment. Histone methylation and acetylation marks [30] were downloaded from the SRA repository - [SRA:SRA000206] and [SRA:SRA000287], respectively. Raw sequences were aligned on the human genome assembly (NCBI36) using the Soap 2.01 alignment tool with default options; we only considered exact matches. To evaluate if the strength of the periodicity and the intensity of the acetylation are correlated (Figure S4 in Additional file 1), we computed the distribution of tag counts in the +40 to +200 region after the TSS for the six histone marks linked to EP300 (see above), for the 12,270 sequences that possessed at least one tag. The distribution was divided into quartiles, the RR periodicity at 10 bp was computed for each quartile and a Pearson correlation test was performed between tag count and magnitude of the periodicity at 10 bp. The H3K56 acetylation data [32] consist of ChIP-chip results on a 244K Agilent Human promoter microarray using immunoprecipitated DNA sequences bound to H3K56 acetylated nucleosomes. Of the 13,622 TSSs used in our study, 6,518 possessed at least one 244K microarray probe positioned between +40 and +200 bp after the TSS in the region overlapping the +1 nucleosome.

### Gene expression and Gene Ontology analyses

Human gene expression data from the HG-U133A and GNF1B Affymetrix chips were obtained from the Genomics Institute of the Novartis Research Foundation [46]. After filtering and remapping of probes (Supplementary information in Additional file 1) we obtained 4,372 genes that were also present in the dbTSS dataset and were used for the analysis. The distribution of the median of the normalized expression levels [47] across the 72 tissues for each gene showed a bimodal distribution that we partitioned using a Gaussian mixture model (Figure S6 in Additional file 1). The two sets of low (L; 1,846 genes) and high (H; 2,526 genes) expression level were analyzed by randomization tests (see below). The quantification of tissue specificity is described in Supporting information in Additional file 1. The distribution of the tissue specificity scores for the 4,372 genes was divided into 3 groups containing 1,199, 2,159 and 1,014 genes (Figure S7 in Additional file 1) with low, medium and high tissue specificity levels, respectively, and the periodicity was measured for each group by bootstrapping as described for the median expression level. The enrichment of Gene Ontology terms in the H versus the L groups was performed using FatiGO+ software [48], available through the Babelomics site [49].

### EP300 binding

The 7-bp consensus sequence binding site of EP300 [28] (matrix from Transfac [M00033] release 7; Figure S11 in Additional file 1) was searched between positions +1 and +40 after the TSS using the position specific weight matrices method [50]. We computed a local probability score using a sliding window of 7 bp (that is, the consensus size) from -500 bp to +500 bp around the TSS. A match for a putative EP300 binding site occurs if the local probability is higher than the average probability computed along the region (probability density estimation [51]). A total of 198 sequences have a unique match located between 0 and +40 bp after the TSS, and these were used for further analysis. To further account for possible compositional biases in this region, we performed multiple random shuffling of the position within the matrix and computed the distribution of occurrences in the same region as above. None of the iterations generated a significant number of matches in the 0 to +40 region, confirming that the 198 sequences are highly enriched in specific matches to the EP300 position weight matrix.

### Randomization tests

In several steps of the analysis described here, we wished to test the strength of the PSD magnitude at 10 bp (see 'Power spectral analysis' section above) on a set of TSS sequences that share a specific property (for example, low gene expression level, EP300 binding, and so on). Because calculating a single average PSD value for the whole set of TSSs that share a given property does not provide any means to calculate statistical significance, we performed random sampling with replacement of a subset of TSSs from this population, and calculated PSD values from each sample based on its average RR frequencies as a proxy for both RR and YY frequencies (the 'RR/YY signal'). The distributions obtained in this way are normal, and can be compared to assess if they are statistically different between two populations of sequences. The size of each random sample used here is composed of between 500 and 1,000 sequences (depending on the initial size of the promoter group). The number of samplings required to reach a normal distribution (*P*-value < 1 × 10^-5^, Kolmogorov-Smirnoff test) is between 2,000 and 5,000.

## Abbreviations

bp: base pair; ChIP: chromatin immunoprecipitation; ChIP-seq: ChIP with DNA sequencing; DFT: discrete Fourier transform; PSD: power spectral density; RNA pol II: RNA polymerase II; RR dinucleotide composed of purine bases (A or G); SRA: Short Read Archive; TSS: transcription start site; WW: dinucleotide composed of A or T; YY: dinucleotide composed of pyrimidine bases (C or T).

## Authors' contributions

CH performed the analyses and participated in the design of the study. HRC conceived the study and wrote the manuscript. All authors read and approved the final version of the manuscript.

## Supplementary Material

Additional file 1 **Supplementary information and Tables S1 and S2 and Figures S1 to S11**. This file contains additional details on CpG classification, correlation between CpG dinucleotides and periodicity, comparisons between promoters with and without CpG islands, methods for quantifying gene expression and tissue specificity, and the relationship between transcription factor binding site periodicity and dinucleotide periodicity. Click here for file
