# Supplementary material for: Nucleosome rotational setting is associated with transcriptional regulation in promoters of tissue-specific human genes
Source: Genome Biol. 2010 May 12;11(5):R51. doi: 10.1186/gb-2010-11-5-r51 (PMC2898081; doi:10.1186/gb-2010-11-5-r51)
Supplement: Additional file 1 — Supplementary information and Tables S1 and S2 and Figures S1 to S11. This file contains additional details on CpG classification, correlation between CpG dinucleotides and periodicity, comparisons between promoters with and without CpG islands, methods for quantifying gene expression and tissue specificity, and the relationship between transcription factor binding site periodicity and dinucleotide periodicity. [file gb-2010-11-5-r51-S1.DOC]

**Nucleosome rotational setting is associated with transcriptional regulation in promoters of tissue specific human genes**

Charles HEBERT and Hugues ROEST CROLLIUS

*Institut de Biologie de l’Ecole Normale Supérieure (IBENS), CNRS UMR8197, INSERM U1024, 75005 Paris, France.*

**1°) CpG classification**

The CpG classification is based on the normalized CpG content measured in a 3kb window centred on the TSS. Normalized CpG fraction was computed as (observed CpG)/(expected CpG), where expected CpG was calculated as (GC content/2)^2^, as described in reference 26. The threshold is defined by the Gaussian intersect (Normalized CpG fraction = 0.35). We obtained 9644 (70.8%) CpG-containing promoters, and 3878 (29.2%) non-CpG-containing promoters (Supplementary Figure 2).

#### 2°) Within CpG-island containing promoters, the periodic signal is not correlated to the overrepresentation of CpG dinucleotides

Because the 10-bp periodic signal is present exclusively with the group of promoters that contain CpG islands, it is possible that within this group of promoters the degree of CpG overrepresentation is correlated with the strength of the periodic signal. To evaluate this possibility we partitioned the normalized CpG content distribution of the 9644 promoters with CpG islands into 5 quantiles and computed the normalized DFT periodogram for each quantile (see methods in main text). The correlation between the normalized magnitude at 10 bp and the normalized CpG content is not significant (Pearson’s correlation = 0.14; p-value = 0.8145).

**3°) Comparison of dinucleotide profiles between promoters with and without CpG islands.**

We analyzed the frequencies of the 16 possible dinucleotides using Discrete Fourier Transform in the two sets of promoters: with and without CpG islands. We normalized the amplitude of the 10 bp period against the sum of all amplitudes arising between 5 and 20 bp (Figure S3) and, for each dinucleotide we subtracted the resulting amplitude obtained for the 3,978 promoters without CpG islands from the amplitude obtained for the 9,644 CpG containing promoters (Supplementary Table 1). This provides, for each dinucleotide, a score reflecting its differential periodicity profile between the two sets of sequences.

**4°) Gene expression data and quantification of tissue specificity**

Human gene expression data from the HG-U133A and GNF1B Affymetrix chips were obtained from the Genomics Institute of the Novartis Research Foundation. Cancerous tissues and probes with hits in more than one human gene coding sequences were removed from the analysis. The expression data set eventually consisted of 11774 and 17712 probes for the U133A and GNF1H chips respectively, tested with 72 tissues. The data was normalized with the gcRMA method (reference 47) and the probes mapped to Ensembl (version 53) predicted coding sequences, resulting in 4,665 genes with expression data. Of these, 4,372 genes were also present in the dbTSS dataset and were used for the analysis.

To quantify tissue specificity we computed for each gene its relative expression in a particular tissue:


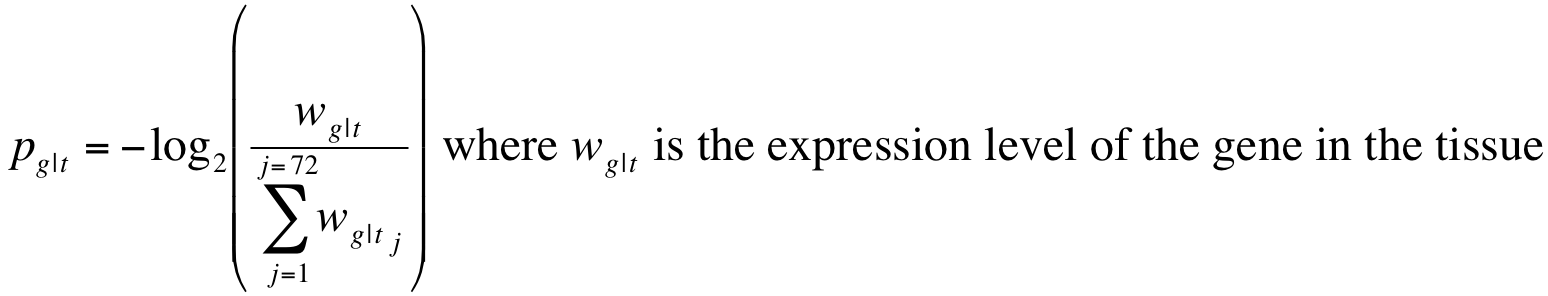


Normalized expression levels ranged from 12.9 to 535022.6 (arbitrary units). A gene expressed at similar levels in all tissues will have similar *p_g|t_* values for all tissues. A gene expressed exclusively in one tissue will have *p_g|t_*=0 for that tissue. Then for each gene we defined the tissue specificity score as the minimum ratio between each p_g|t_ values and the average p_g|t_ value for all tissues:


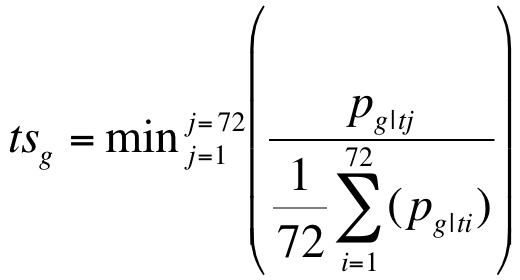


Ubiquitously expressed genes, with similar *p_g|t_* values across all tissues, will always show a ratio close to 1, and thus the minimal ratio *ts_g_* will also be close to 1.

#### 5°) Transcription factor binding site periodic occurrences are an indirect *consequence* of the periodic RR and YY signal.

The AA/TT/TA/AT periodic signal observed in yeast has already been proposed to act as a rotational cue when the histone core is wrapped by the 147 bp of DNA. Instead of promoting a favorable spatial arrangement between different molecular complexes as described by our model, the model by Alberts et al. proposes that the rotational setting exposes transcription factor binding sites to the external face of the nucleosome, thus making them available for binding. This is supported by the observation that conserved binding sites tend to concentrate in 10 bp periodic positions that align with the major groove of the DNA, presumably because these positions are counter phase with the AA/TT/AT/TA dinucleotide frequency peaks.

We therefore examined if similar observations could be made in the human genome. Indeed, although we observed the periodic RR and YY signal in human genes exclusively downstream of the TSS coinciding with the +1 nucleosome, it remains a possibility that functional human TF binding sites, that are known to occur anywhere in the genome, may display the same periodic pattern in human +1 nucleosome as in yeast -1 nucleosome.

We obtained Position Frequency Matrices (PFM) for 153 human TF binding sites from the Transfac database (v7, 2005). Each PFM was transformed in a Position Weighted Matrix (PWM) using the RSATools method (RSAT: regulatory sequence analysis tools. Nucleic Acids Res. 2008 Jul 1;36), using a prior background frequency equal to 0.25 for each nucleotide. For each PWM, we then computed a score at each base pair position downstream of the 13,622 TSS sequences that constitute our TSS set, and took the average of the scores at each position. An example is shown in Figure S10A. We performed on these signals a power spectral density analysis (see methods in main text) and obtained 153 periodograms. Each periodogram thus shows, for a given TF binding site, the periodicities that may exist in the distribution of its occurrences in the region bound by the +1 nucleosome. Strikingly, most TF binding sites show a distinct 10 bp periodicity. Figure 9A shows the sum of the periodograms for the 20 matrices that show the strongest periodic pattern.

Two scenarios may explain this observations: (i) the region between +40 and +200 downstream of TSSs truly contain regular occurrences of TF binding sites, as shown in yeast, and this is independent of the existing RR and YY dinucleotide period pattern, i.e. the sequence information related to the TF binding sites is carried by different bases than those responsible for the RR/YY periodicity (ii) the TF binding sites are in fact non specific and their periodic occurrence is simply due to the known RR/YY periodicity. In this scenario, TF binding sites that contain motifs that are either similar or very different to the RR/YY periodic signal will respectively be over-represented and excluded over RR/YY peaks, and in both case will be periodic.

To distinguish between the two scenarios, we modified the 20 most periodic matrices by randomly shuffling the order of the positions within each matrix. Each new matrix thus contains the same base composition as the reference matrix but in a different (random) order and will in principle match entirely different sites. The new matrices were used to scan the 13,622 TSSs as described above. The resulting average periodogram, shown in figure S9B, indicates that the new matrices are still highly periodic in the region +40 to +200, despite being partially randomized. To further eliminate any biological information from the matrices, we randomly shuffled both the position and the weights in each matrix, and their resulting periodicity is shown in figure 9C. The average score is still highly periodic, demonstrating that the 10 bp TF binding site periodicity is not related to the sequence information carried by the PWM. The only remaining possibility is that TF binding sites occurrences (and in fact, any random motif) are periodic as a secondary effect of the presence of the RR and YY periodicities. Figure S10 illustrates our approach for the most periodic TRANSFAC matrix M000155.

**Supplementary Table 1.** Dinucleotide differential score at 10 bp between promoters with and without CpG islands. In bold are shown the four dinucleotides with the highest score, GA and AG purine-purine dinucleotides, and their complementary versions CT and TC pyrimidine-pyrimidine dinucleotides.

| Dinucleotide | Differential Score | Rank |
| --- | --- | --- |
| **GA** | **12.7** | **1** |
| **CT** | **7.2** | **2** |
| **TC** | **5.7** | **3** |
| **AG** | **5.4** | **4** |
| GC | 5.3 | 5 |
| GG | 5.2 | 6 |
| CA | 4.5 | 7 |
| CC | 4.5 | 8 |
| GT | 4.3 | 9 |
| TG | 2.5 | 10 |
| TT | 2.0 | 11 |
| AA | 0.5 | 12 |
| TA | 0.5 | 13 |
| AC | 0.5 | 14 |
| AT | -0.4 | 15 |
| CG | -0.5 | 16 |

**Supplementary Table 2.** Gene Ontology enrichment in tissue specific genes that show increased periodic signal

| GO level | GO id | GO term | % in group H | % in group L | Adjusted  p-value |
| --- | --- | --- | --- | --- | --- |
|  |  |  |  |  |  |
| ***Biological process*** | | | | | |
| 5 | GO:0016070 | RNA metabolic process | 30.95 | 10.74 | 7.37e-18 |
|  | GO:0006350 | Transcription | 28.62 | 10.24 | 7.45e-16 |
| 6 | GO:0032774 | RNA biosynthetic process | 29.67 | 10.50 | 5.85e-15 |
|  | GO:0019219 | Regulation of nucleobase, nucleoside, nucleotide and nucleic acid metabolic process | 30.64 | 11.46 | 1.33e-14 |
| 7 | GO:0006351 | Transcription, DNA-dependant | 35.32 | 13.04 | 3.01e-14 |
|  | GO:0045449 | Regulation of transcription | 13.04 | 13.91 | 2.13e-13 |
| 8 | GO:0006355 | Regulation of transcription, DNA-dependant | 37.44 | 14.26 | 2.70e-13 |
| ***Molecular function*** | | | | | |
| 3 | GO:0003676 | Nucleic acid binding | 30.10 | 11.97 | 2.96e-17 |
| 4 | GO:0003677 | DNA binding | 26.09 | 10.15 | 8.43e-13 |
| 5 | GO:0046914 | Transition metal ion binding | 36.72 | 18.29 | 6.00e-11 |
| 6 | GO:0008270 | Zinc ion binding | 38.16 | 17.25 | 2.94e-11 |
| ***Cellular component*** | | | | | |
| 8 | GO:0005634 | Nucleus | 58.6 | 35.88 | 3.07e-10 |

Note: (p-values > 10^e-10^ not shown)


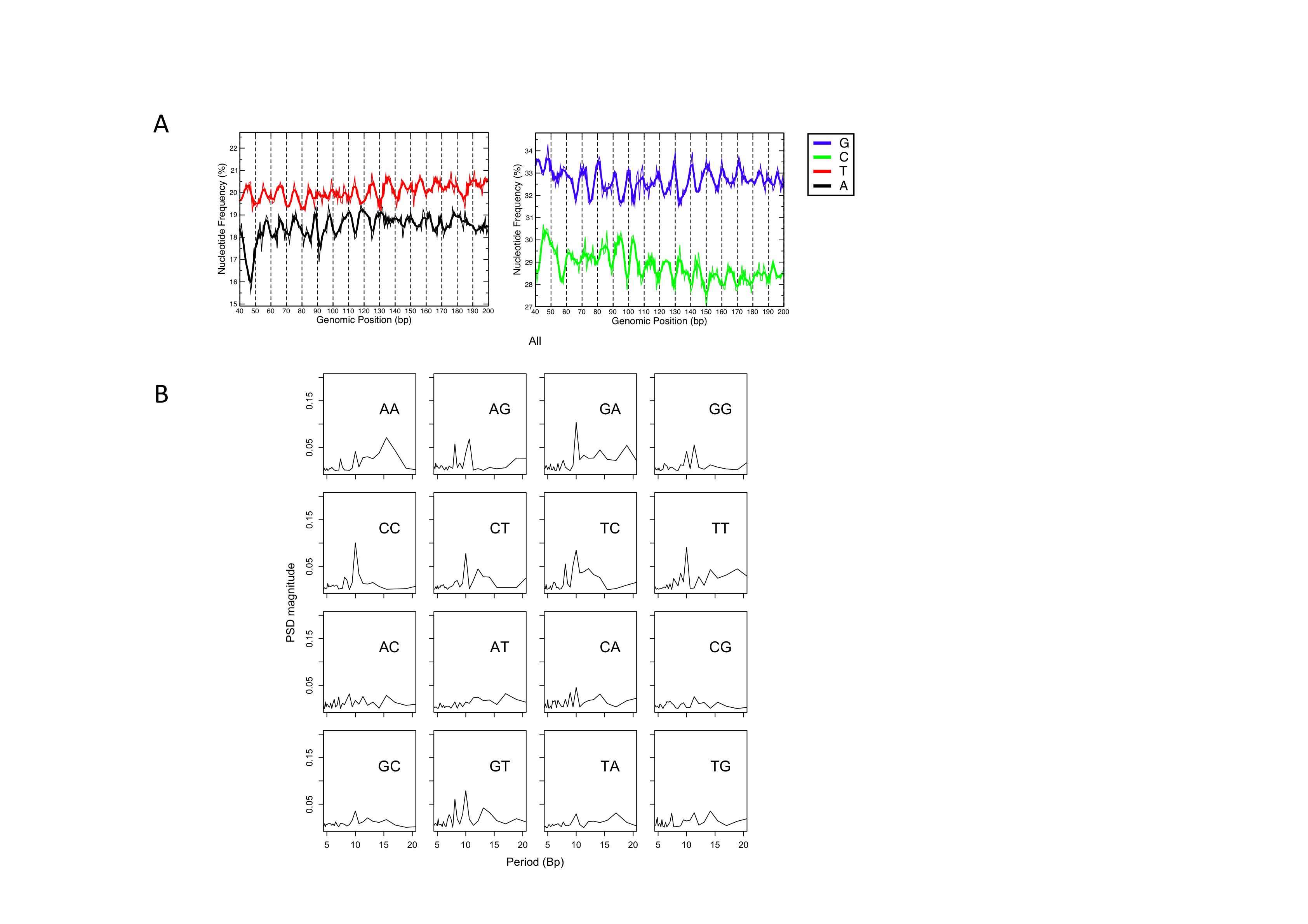


#### Supp Fig. 1:

(A) Average single nucleotide frequency profiles in the region from +40 to +200 after the TSS, computer over 13622 human sequences. This figure is equivalent to a zoom on figure 1 in the main text, at the above position. A periodicity of ~10 bp is evident. (B) Panel of 16 average dinucleotide periodograms computed by Discrete Fourier Transform across the region +40 to +190 after the transcription start site of 13622 human promoters. All periodograms are normalized to allow for comparisons (see methods in main text).


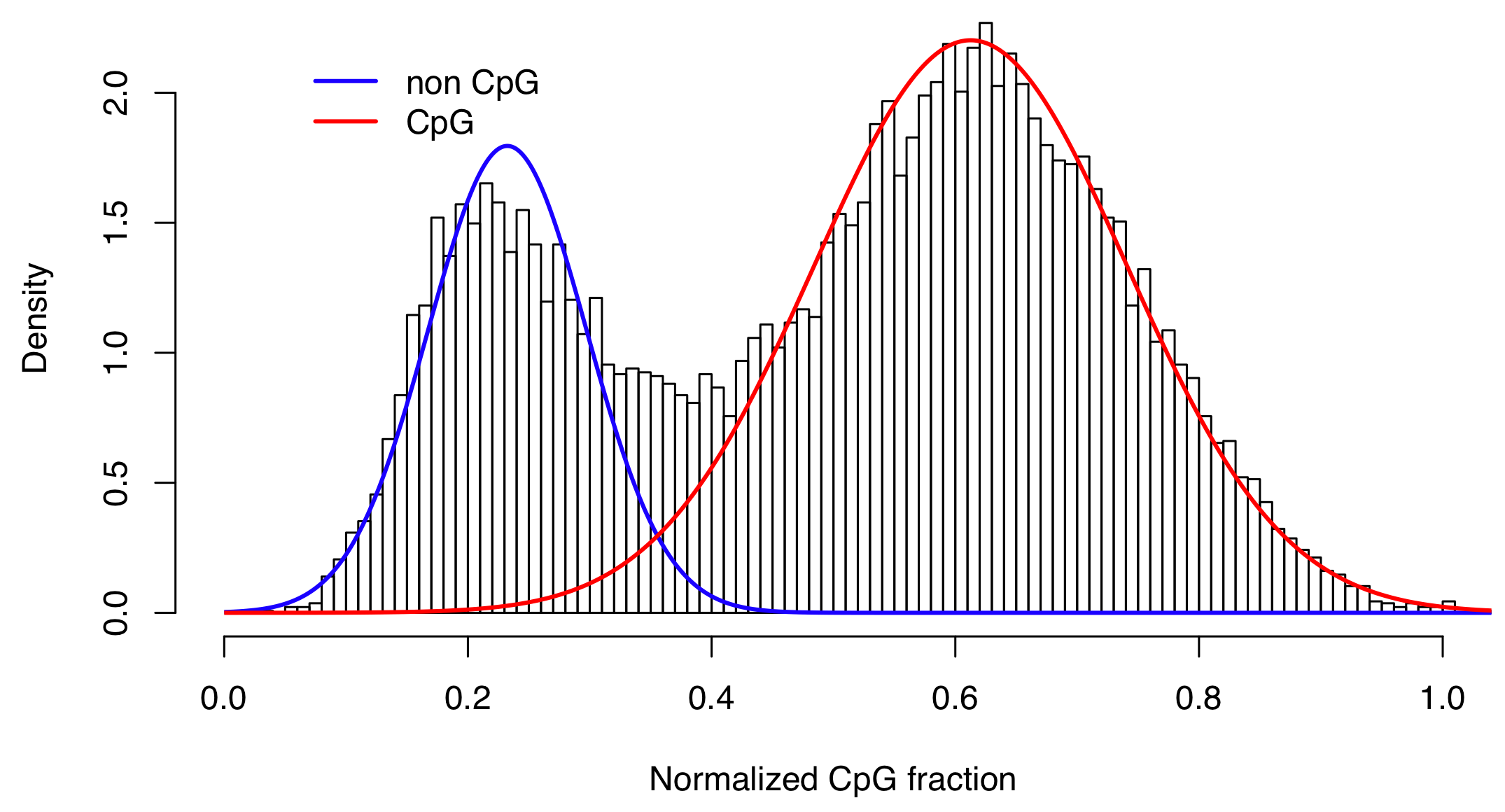


#### Supp Fig. 2:

Parameterized Gaussian mixture model describing the distribution of normalized CpG content in 13,622 human promoter sequences. For each sequence, a 1.5 kb region on either side of the TSS was used to compute the normalized CpG content (see methods), thus defining two Gaussian distributions that separate at the intersect (normalized CpG content = 0.35) a group of 9644 promoters that contain CpG islands from a group of 3978 promoters that do not.


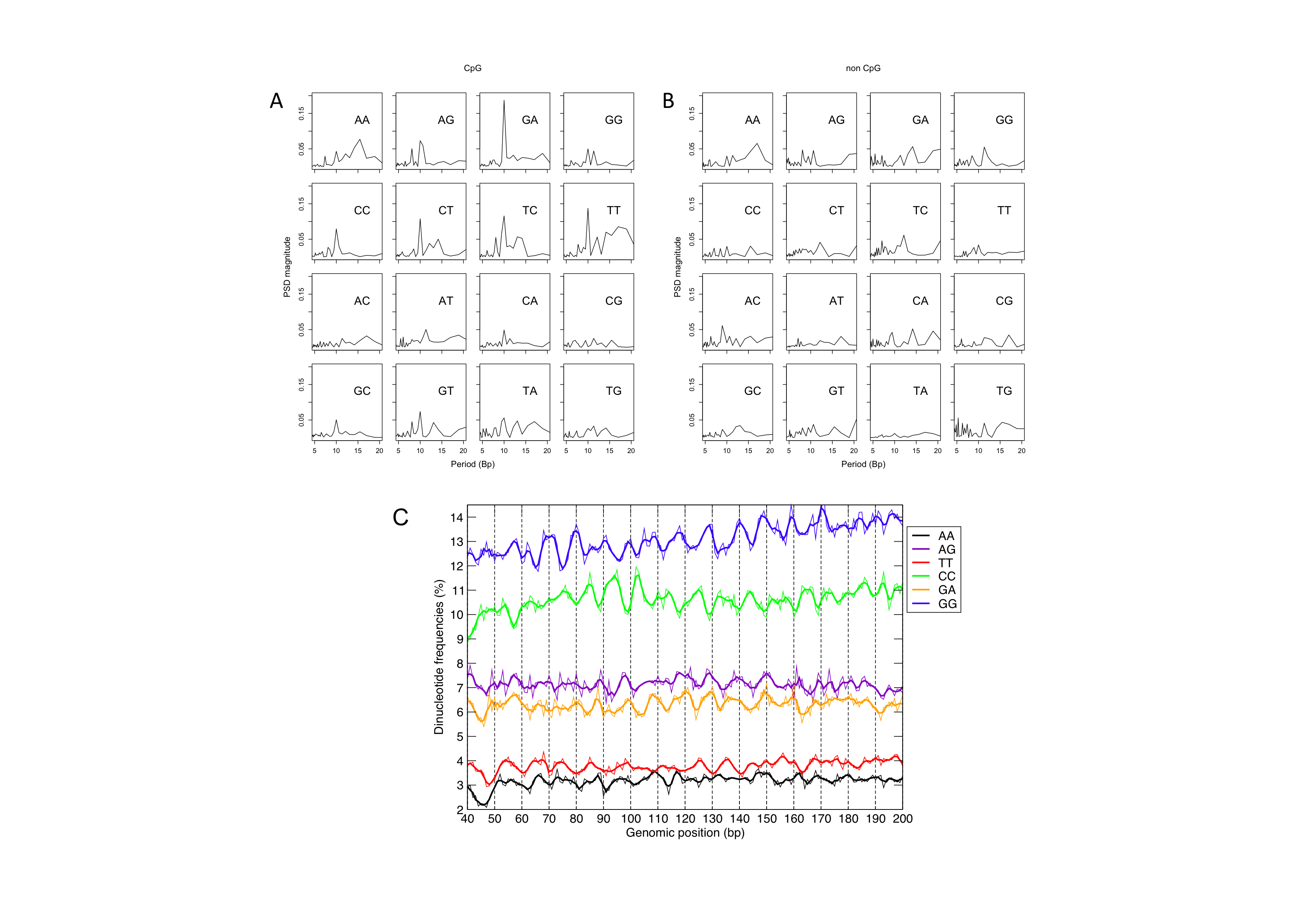


#### Supp Fig. 3:

(A) Panel of 16 dinucleotide frequency spectra computed by DFT across the region +40 to +190 of 9,644 after the TSS of human promoters with CpG islands. (B) same as in A but on 3,978 promoters without CpG islands. (C) Average dinucleotide frequency plots for specific dinucleotides in the region +40 to +200 after the TSS of 9,644 sequences with CpG islands.

**
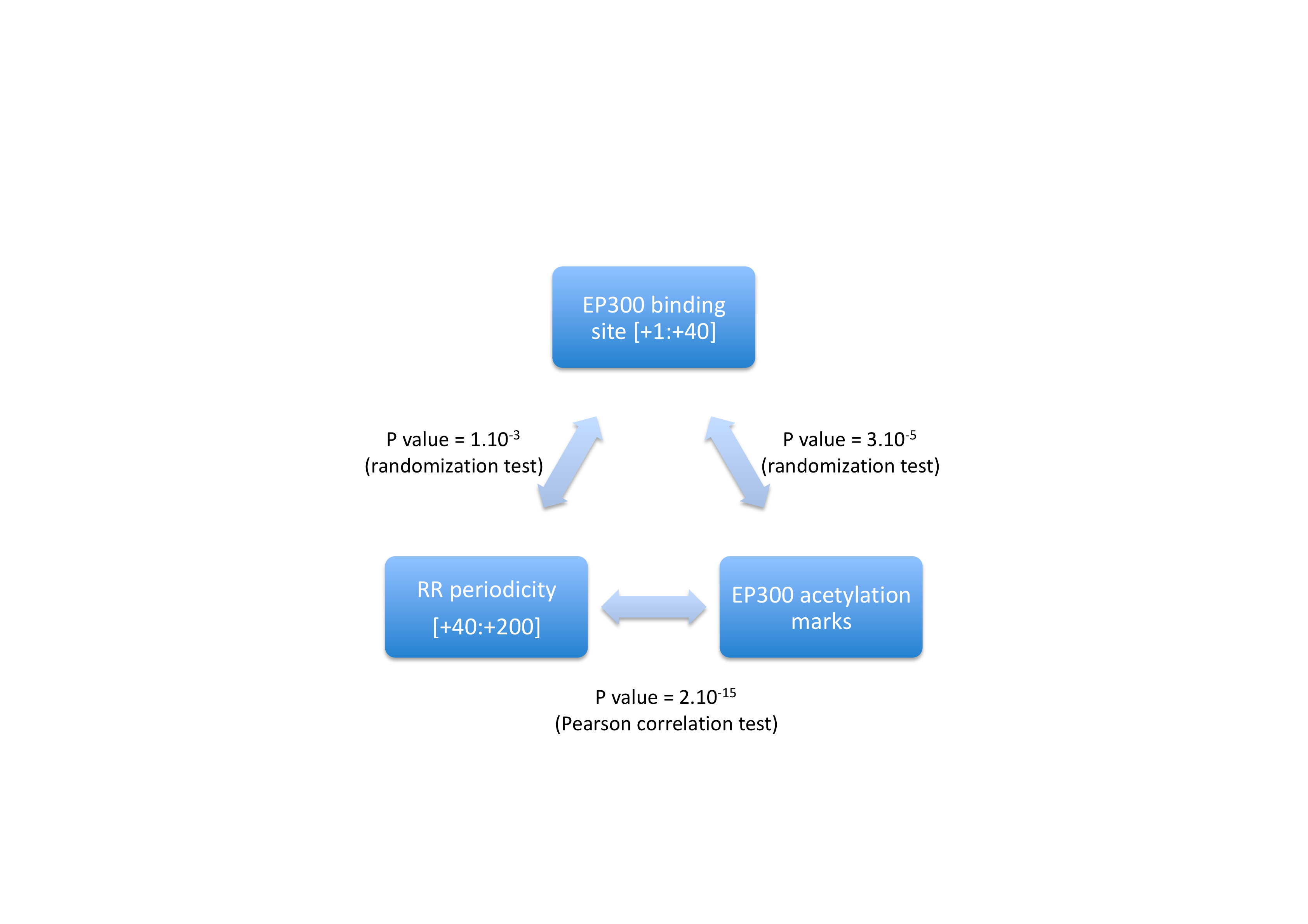
Supp Fig. S4.**

Correlations between the EP300 binding site, the periodicity, and EP300 acetylation in human promoters. The top box represents 198 sequences that possess a significant match of the EP300 binding site (Transfac matrix M0033, see Methods) in the +1 bp to +40 bp region after the TSS. The bottom right box represents the set of 12770 sequences that contain at least one sequence tag characteristic of one of the 6 histone marks deposited by EP300 (see Methods). The bottom left box represents the action of computing the periodicity of RR dinucleotides in the +40 to +200 region after the TSS. The randomization test between the EP300 binding site and the RR periodicity consisted in drawing 10000 random samples of 198 sequences among the 13622 available promoter sequences, and computing the magnitude of their RR periodicity at 10 bp in the region +40 to +200. The magnitude of the periodicity measured for the 198 sequences containing EP300 binding sites was higher than random samples in 99.9 % of the case, thus providing an empirical P-value of 1.10^-3^. The randomization test between the 198 sequences containing EP300 binding sites and the 12770 sequences containing EP300 acetylation marks consisted in the same test as above but this time counting the number of ChIP-seq sequence tags corresponding to the EP300 marks instead of computing the RR periodicity. The number of sequence tags on the 198 sequences containing the EP300 binding sites was higher in 99.997 % of the cases, thus providing an empirical P-value of 3.10^-5^. The Pearson correlation test is explained in details in the Methods section of the main text.


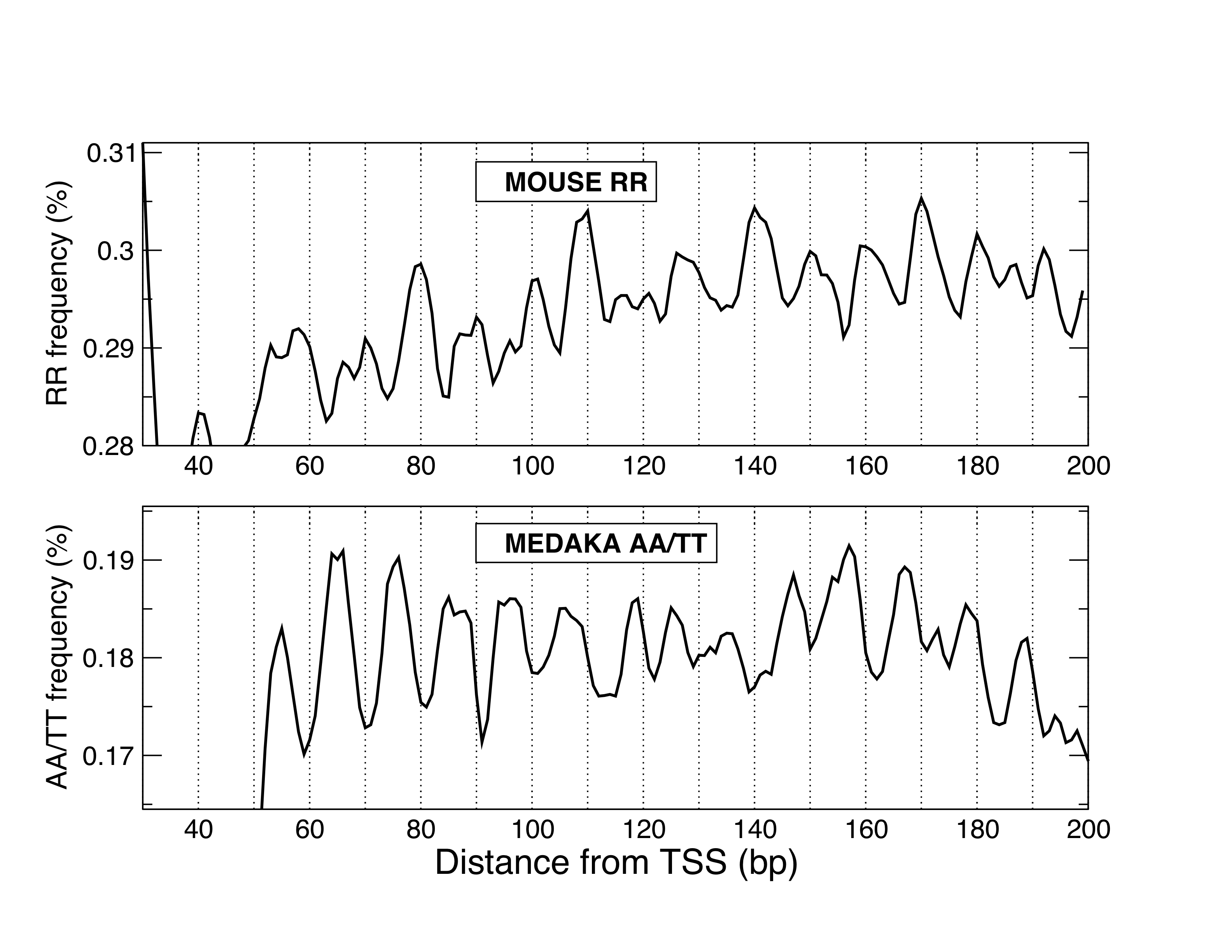


#### Supp Fig. S5.

Periodic dinucleotide frequency in mouse and medaka promoters **a**. In mouse, the same RR and YY dinucleotides are periodic as in human. Mouse promoters were extracted from dbTSS and filtered using the same procedure as for human TSSs, resulting in 17,071 TSSs. **b**. in the medaka fish *Oryzias latipes*, the periodic signal is contributed by AA and TT dinucleotides, similar to the yeast genome.


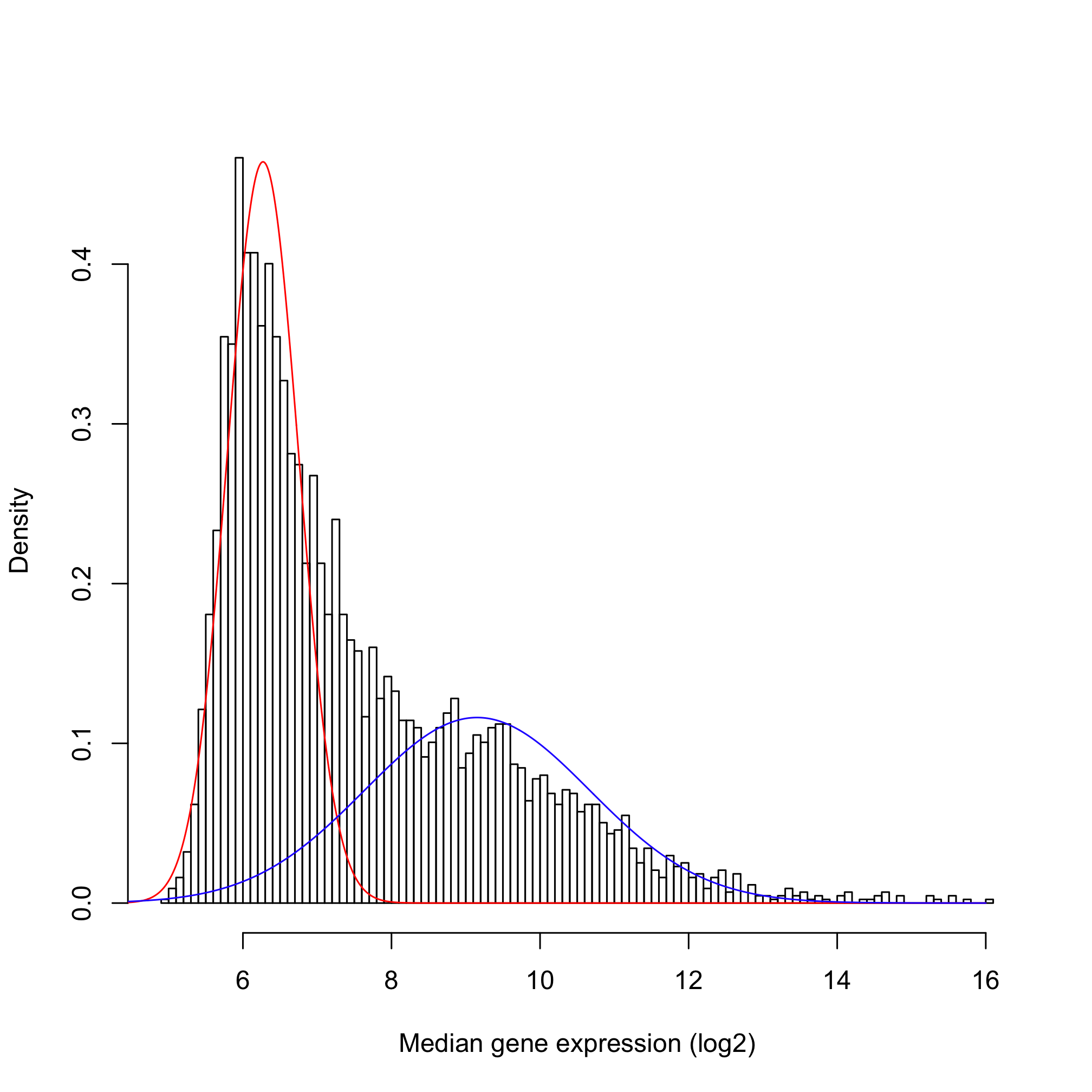


#### Supp Fig. S6.

Partition of human genes into low (red) and high (blue) expression sets. The distribution of the normalized median expression (log_2_) is shown for 4,365 human genes. A Gaussian mixture estimation uncovers two underlying distributions used to partition the data at the intersection (threshold = 7.44) in order to test for RR periodicity.


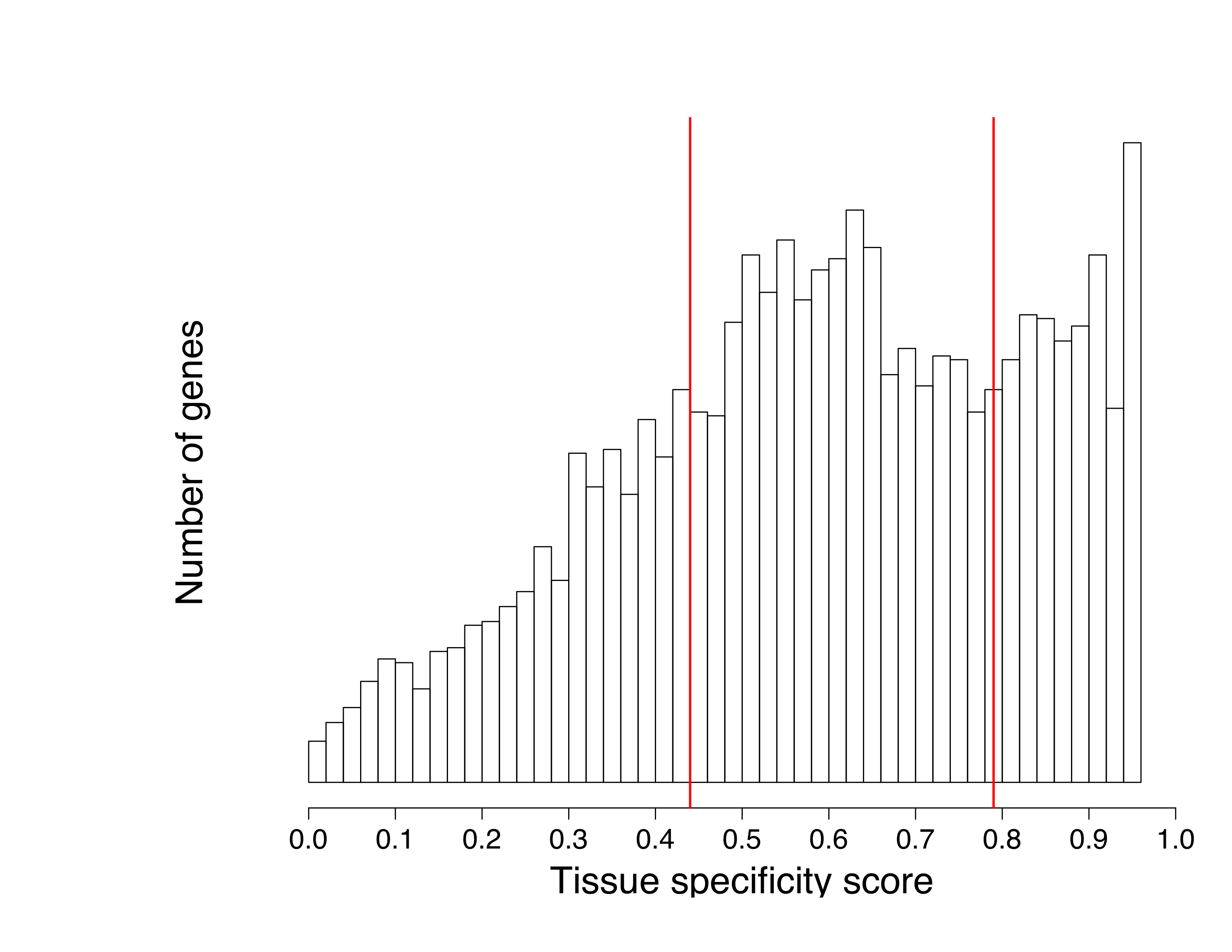


#### Supp Fig. S7.

Distribution of tissue specificity score *t_g_* (see Methods) for 4,372 human genes, with the arbitrary chosen partition thresholds shown in red, respectively at *t_g_*=0.44 and *t* =0.79, thus defining three groups H (1,014 genes), M (2,159 genes), and L (1,199 genes). Note that high tissue specificity is indicated by a low score.


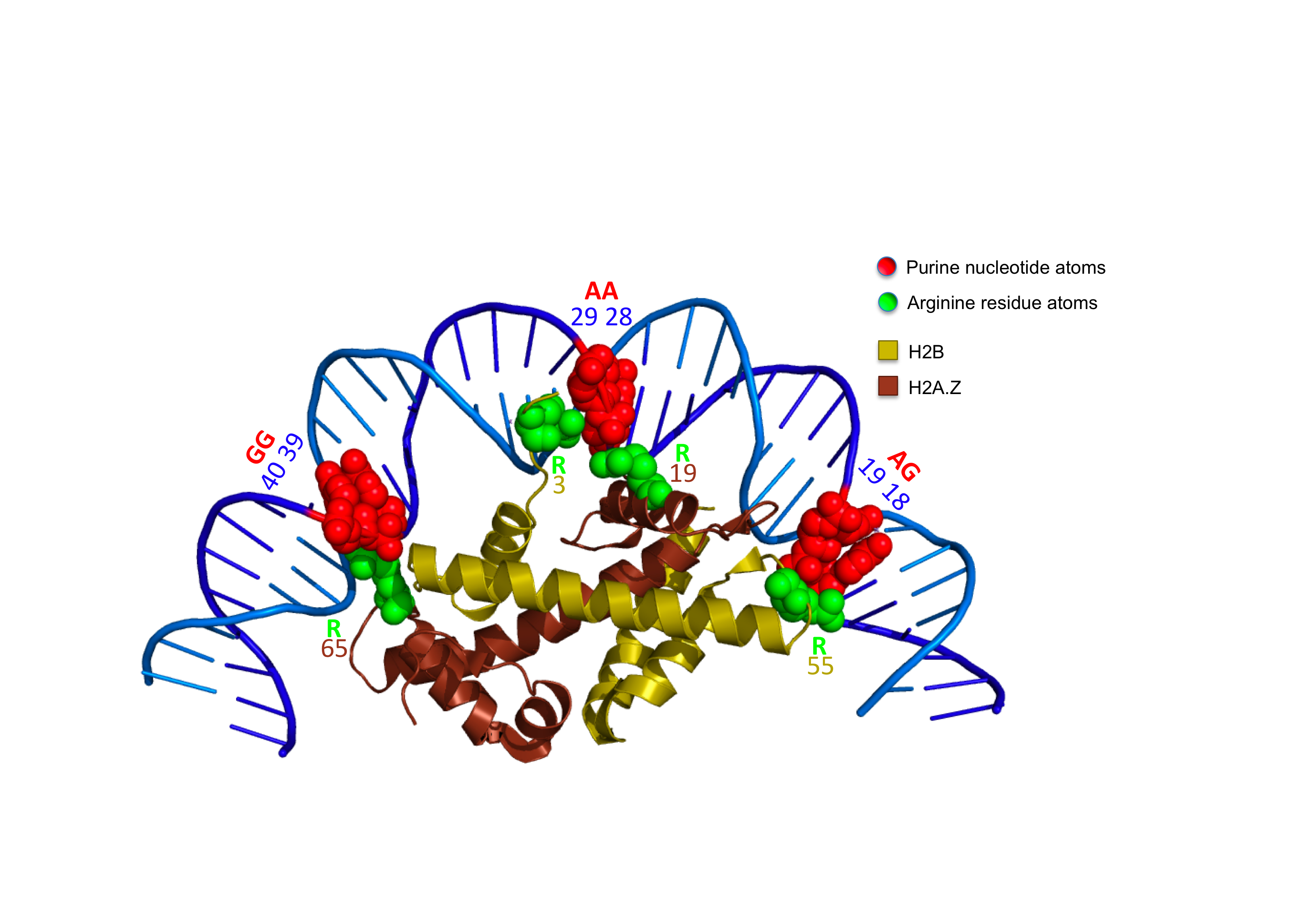


#### Supp Fig. S8.

**Detail of the structure of the H2A.Z nucleosome (PDB accession: 1f66).** The first 50 base pairs of the DNA molecule are shown (blue) with the atoms of some purine-purine (RR) dinucleotides located in three successive histone:DNA contact points in the minor groove of the double helix (red). Numbers in blue indicate the base pair position of the nucleotides on the DNA. For clarity, only the H2B and H2A.Z histones are shown, which are those interacting with the DNA molecule in this region. Four arginine (R) residues are within close range of the RR (GG,AA,AG) dinucleotides (green). The figures in brown or gold indicate the position of the arginine on their respective protein sequence.


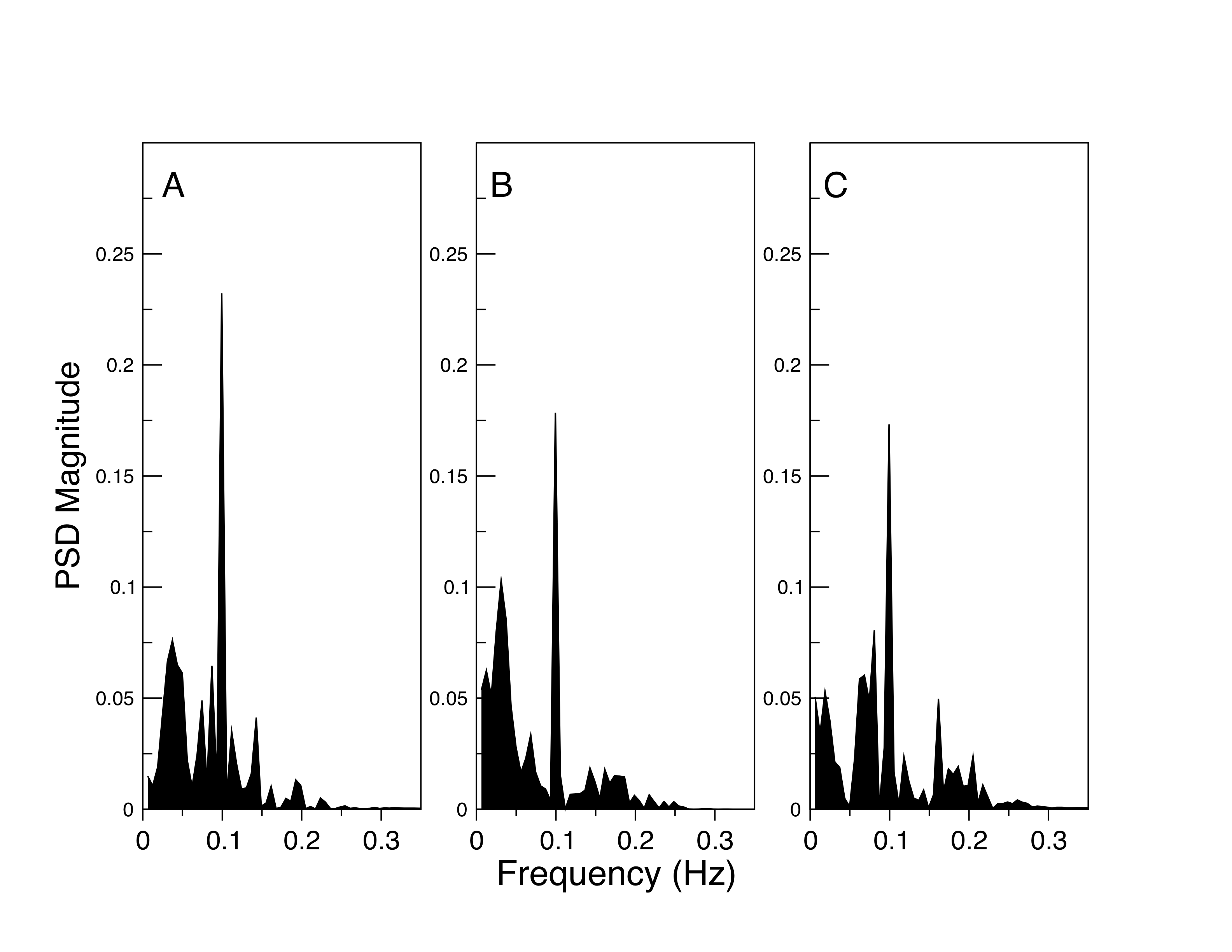


#### Supp. Fig. S9.

Average periodograms of 20 Transfac matrices showing the highest individual 10 bp periodic scores. (A) Periodogram for the original matrices, showing a strong peak at 0.1 Hz indicative of a 10 bp periodicity of the PWM scores in the region +40 to +200 after the TSS. (B), same as in A but where the positions within each matrix were randomly shuffled. (C) same as in A but where both the positions and the weights within each matrix were randomly shuffled to erase any remaining biological signal. The fact that these randomized matrices generate a strong 10 bp periodicity demonstrates that the latter is independent of the biological signal carried by the matrices. The Transfac accession numbers of the 20 most periodic matrices are: M00025, M00037, M00039, M00052, M00053, M00054, M00059, M00121, M00156, M00155, M00158, M00173, M00185, M00186, M00187, M00188, M00191, M00205, M00215, M00224. The individual case of matrix M00155 is illustrated in figure S10.


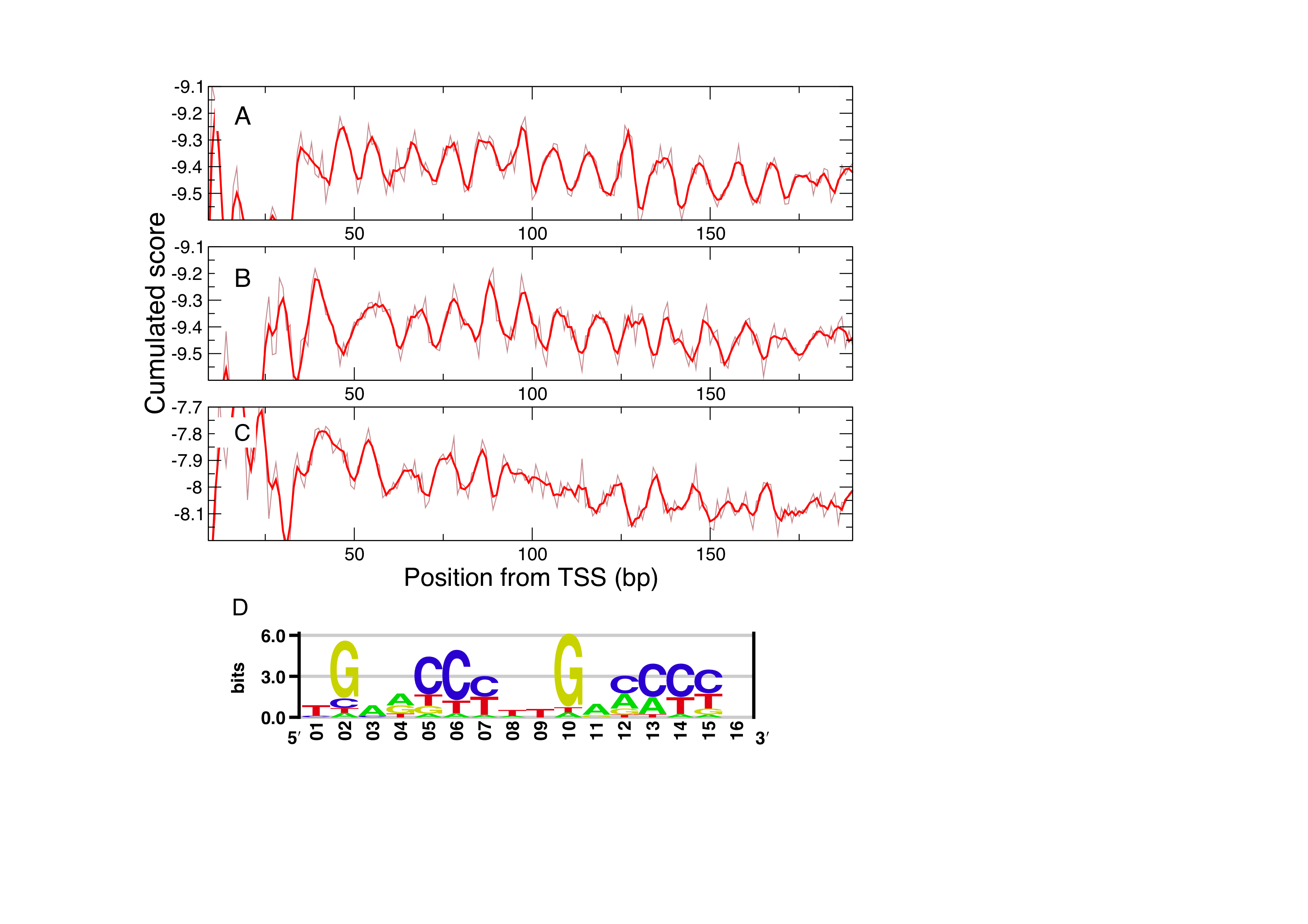


#### Suppl. Fig. S10.

Periodicity of the PWM for matrix M00155, corresponding to the transcription factor Apolipoprotein AI regulatory protein 1 (ARP-1). (A). Average score of the PWM obtained on 13,622 sequences comprised between +40 and +200 bp downstream of the TSS of human genes. (B) the PWM scores of a matrix of same size and same base composition as M00155 but where the order of the positions within the matrix was randomly shuffled (C) the PWM scores of a matrix of same size as M00155 but where both the positions and the weights were shuffled. (D) the sequence logo of the original M00155 matrix. While the signal in A is periodic, the matrix in B is still periodic despite it representing an entirely different sequence than A (but of same base composition and frequencies). Even the periodogram in C shows a residual periodicity despite the fact that both base positions and base frequencies were randomly shuffled. This hierarchy is immediately explained by the sequence logo of the original M00155 matrix indicated in D, where two pyrimidine nucleotides (G) show high frequencies at position 2 and 10, and are interleaved (with a 5 bp shift) with two purines (C/T) clusters separated by 9-nucleotides. As shown in A, this motif is likely to elicit a strong periodic pattern of occurrences in sequences that are known to contain a periodic purine-purine pattern interleaved with a pyrimidine-pyrimidine pattern shifted by 5 bp.


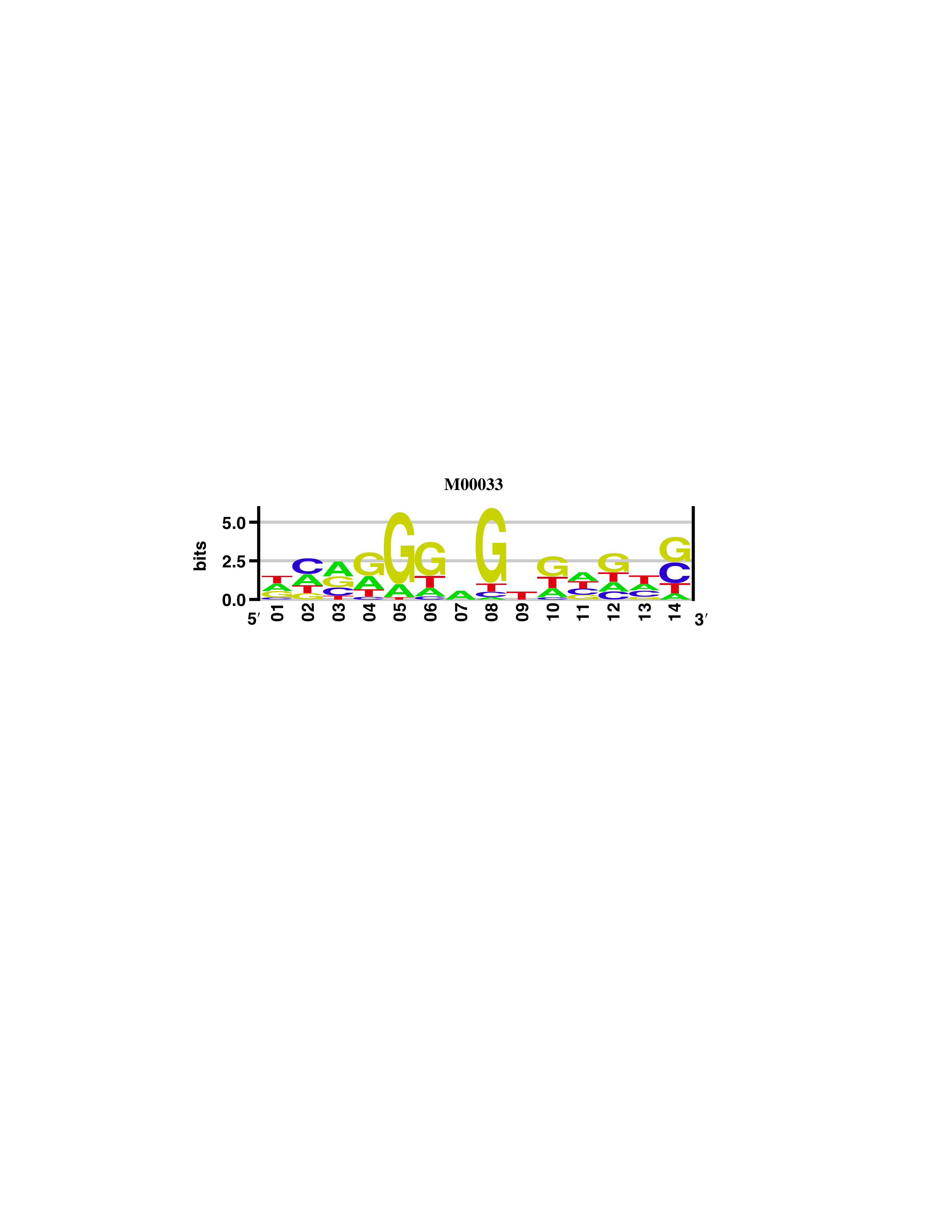


**Suppl. Fig. S11.**

Sequence logo for the EP300 binding matrix described in reference 28 and available from the Transfac (public version 7) database under accession number M00033. The consensus sequence used to compute the positions of the matrix in human sequences is restricted to the 7 positions 04 to 10 in the logo.
